# Analysis of *HLA* Variants and Graves’ Disease and Its Comorbidities Using a High Resolution Imputation System to Examine Electronic Medical Health Records

**DOI:** 10.3389/fendo.2022.842673

**Published:** 2022-03-07

**Authors:** Wen-Ling Liao, Ting-Yuan Liu, Chi-Fung Cheng, Yu-Pao Chou, Tzu-Yuan Wang, Ya-Wen Chang, Shih-Yin Chen, Fuu-Jen Tsai

**Affiliations:** ^1^Graduate Institute of Integrated Medicine, College of Chinese Medicine, China Medical University, Taichung, Taiwan; ^2^Center for Personalized Medicine, China Medical University Hospital, Taichung, Taiwan; ^3^Center for Precision Medicine, China Medical University Hospital, Taichung, Taiwan; ^4^Genetic Center, Department of Medical Research, China Medical University Hospital, Taichung, Taiwan; ^5^Department of Laboratory Medicine, China Medical University Hospital, Taichung, Taiwan; ^6^Division of Endocrine and Metabolism, China Medical University Hospital, Taichung, Taiwan; ^7^School of Chinese Medicine, China Medical University, Taichung, Taiwan

**Keywords:** genome-wide association study (GWAS), phenome-wide association studies (PheWAS), electronic medical record (EMR), human leukocyte antigen (HLA), Graves’ disease (GD)

## Abstract

Hyperthyroidism is a prevalent endocrine disorder, and genetics play a major role in the development of thyroid-associated diseases. In particular, the inheritance of HLA has been demonstrated to induce the highest susceptibility to Graves’ disease (GD). However, thus far, no studies have reported the contribution of HLA to the development of GD and the complications that follow. Thus, in the present study, to the best of our knowledge, for the first time, a powerful imputation method, HIBAG, was used to predict the HLA subtypes among populations with available genome-wide SNP array data from the China Medical University Hospital (CMUH). The disease status was extracted from the CMUH electronic medical records; a total of 2,998 subjects with GD were identified as the cases to be tested and 29,083 subjects without any diagnosis of thyroid disorders were randomly selected as the controls. A total of 12 HLA class I genotypes (HLA-A*02:07-*11:01, HLA-B*40:01-*46:01 and *46:01-*46:01, and HLA-C*01:02-*01:02, *01:02-*03:04, and *01:02-*07:02) and 17 HLA class II genotypes (HLA-DPA1*02:02-*02:02, HLA-DPB1*02:01-*05:01, *02:02-*05:01, and *04:01-*05:01, HLA-DQA1*03:02, HLA-DRB1*09:01-*15:01, and *09:01-*09:01) were found to be associated with GD in the Taiwanese population. Moreover, the HLA subtypes HLA-A*11:01, HLA-B*46:01, HLA-DPA1*01:03, and HLA-DPB1*05:01 were found to be associated with heart disease, stroke, diabetes, and hypertension among subjects with GD. Our data suggest that several HLA alleles are markedly associated with GD and its comorbidities, including heart disease, hypertension, and diabetes.

## Introduction

Hyperthyroidism is a prevalent endocrine disorder characterized by an inappropriately high synthesis and secretion of the thyroid hormones triiodothyronine (T3) and thyroxine (T4) (1). Graves’ disease (GD) is an organ-specific autoimmune disorder caused by thyroid-stimulatory immunoglobulins and is the most common type of hyperthyroidism (2). The autoantibodies produced imitate the activity of the thyroid-stimulating hormone (TSH) and lead to the stimulation of thyroid function, thus suppressing the TSH levels, while elevating the serum free T4 and T3 levels. Furthermore, thyroid disorders are associated with an abnormal elevation of the levels of serum lipids (3) and are associated with a range of clinical consequences, including an increased risk of metabolic disorders, cardiovascular mortality, and thyroid cancer (4).

Family and twin studies have indicated that genetics plays a major role in the development of thyroid diseases. Many susceptibility loci associated with autoimmunity (human leukocyte antigen [*HLA*], protein tyrosine phosphatase, non-receptor type 22 [*PTPN22*], cytotoxic T-lymphocyte associated protein 4 [*CTLA4*], and interleukin 2 receptor subunit alpha [*IL2RA*]) or thyroid-specific genes (thyroid-stimulating hormone receptor [*TSHR*] and forkhead box E1 [*FOXE1*]) have been identified to be associated with various thyroid diseases (5).

In particular, the inheritance of HLA has been demonstrated to induce the highest susceptibility to GD (6–8). HLA is the most prominent candidate genetic factor for several autoimmune diseases because the major histocompatibility complex region is highly polymorphic and relevant to many immune response genes. The HLA locus is located on chromosome 6p21 and encodes (1): class I genes, such as the *HLA-A, HLA-B*, and *HLA-C*, and (2) class II genes, such as the *HLA-DP, HLA-DQ*, and *HLA-DR* genes (9). According to the IPD-IMGT/HLA Database, there are nearly 8,464 identified *HLA-B* alleles (10). However, HLA-genotyping methods, such as polymerase chain reaction using sequence-specific oligonucleotides or primers, or next-generation sequencing, remain impractical for analyzing large-scale associations.

Nevertheless, to the best of our knowledge, thus far, no study has examined the contribution of HLA to the development of GD and the complications that follow among patients with GD. Hence, the aim of this study was to evaluate HLA variants in GD patients with different complications.

## Material and Methods

### Study Population

The China Medical University Hospital (CMUH) Precision Medicine Project was initiated in 2018, and more than 170,000 subjects have been enrolled in this project to date (5). The recruitment and sample-collection procedures were approved by the ethical committees of CMUH (CMUH107-REC3-058 and CMUH110-REC3-005). In this project, each subject signed an approved informed consent form and provided blood samples for genome-wide genotyping. All clinical information, including disease diagnoses, medical and surgical procedures, prescriptions, laboratory measurements, physiological measurements, hospitalization, and catastrophic illness status were collected from the electronic medical records (EMRs) of the CMUH. The EMRs of CMUH contain the medical records of patients who sought care at the CMUH between 1992 and 2019 (6, 7). This part of the study was approved by the ethical committees of the CMUH (CMUH110-REC1-095).

### Data Source

A total of 2,998 subjects with GD (International Classification of Diseases, 9th Revision, Clinical Modification [ICD-9-CM] 193,194.0-194.9, 240.0-246.9, 376.21 242.90, 242.00, or ICD10-code E05.0), under at least three prescriptions of medications [methimazole (tapazole), propylthiouracil (Procil), or carbimazole (neothyrostate)], with at least one TSH and free T4 or total T4 value, and with genotyping information were identified as subjects with GD (case group). In addition, 29,083 subjects without any diagnosis of thyroid disorders, not using any anti-thyroid/thyroxin medications, and without any abnormal TSH/T4/free values were randomly selected from the EMRs as the control group. Other comorbidities were also identified by ICD-9-CM from the EMRs.

### HLA Genotyping

Human genomic DNA was extracted from the blood samples of all the participants using standardized protocols. The Axiom-Taiwan Precision Medicine (TPM) genotyping SNP array for the high-throughput Affymetrix Axiom genotyping platform was produced to obtain the maximum amount of genetic information from the samples of the Taiwanese population. SNP genotyping was performed using the Axiom-TPM array. A total of 653,291 SNPs across the whole human genome were included in the Axiom-TPM Array Plate (Affymetrix, Inc., Santa Clara, CA, USA).

### HLA Imputation and Prediction

The HIBAG R package prediction algorithm was used to generate Taiwanese population-specific parameter estimates. The estimates were based on individual classifiers consisting of HLA and SNP haplotype probabilities. Probabilities were estimated from the samples and SNP subsets. Finally, the different HLA types were predicted by averaging the posterior probabilities above 0.9 from all the generated classifiers. A total of 16 parameter estimates were generated for two-field and three-field parameter estimates covering HLA-A, C, B, DRB1, DQA1, DQB1, DPA1, and DPB1 (8).

### Statistical Analysis

For the baseline characteristics, continuous data were presented as the means with standard deviation, and categorical data were presented as proportions. We used *t-tests* to compare the mean values of continuous variables and chi-squared tests to compare the frequencies of categorical variables between the two groups. All tests were two-sided, and differences with *P* values < 0.05 were considered statistically significant. Statistical analyses were performed using SPSS software (version 21.0; IBM, Armonk, NY, USA) and R version 3.4.4 (11).

## Results

A total of 2,998 subjects with GD were identified using the EMRs of the CMUH. Demographic and clinical information is shown in **Table 1**. More than 70% of the subjects were women, 66.4% were aged between 30 and 60 years, and 51.8% were obese or overweight. With regard to the comorbidities, 5.4%, 6.6%, and 9.2% of the subjects with GD had hypertension, heart disease, stroke, and diabetes, respectively.

**Table 1:** Characteristics of 2998 Taiwanese patients with Graves’ disease.

Characteristics	
Sex	
Male	637 (21.2%)
Female	2130 (71.0%)
Missing	219 (7.3%)
Age	46.0 (15.62)
<20	80 (2.7%)
20-29	341 (11.4%)
30-39	662 (22.1%)
40-49	712 (23.7%)
50-59	617 (20.6%)
60-69	360 (12.0%)
70+	226 (7.5%)
BMI	25.45 (4.88)
<18.5	70 (2.3%)
18.5-<24	1146 (38.2%)
24-<27	715 (23.8%)
27+	838 (28%)
Missing	229 (7.6%)
Comorbidities	
Thyroid storm	40 (1.3%)
Hypertension	162 (5.4%)
Heart Diseases	103 (3.4%)
Stroke	97 (3.2%)
Hyperlipidemia	45 (1.5%)
Diabetes	276 (9.2%)
Cancer	106 (3.5%)

HLA class I and class II genes were analyzed in subjects with and without thyroid disorders. The allele frequency in controls was similar to that previously reported for the general Taiwan Han population (**Tables S1, S2**) (9, 10). With regard to the HLA class I subtypes (HLA-A, -B, -C), a difference in the distributions for a total of 12 genotypes with a frequency of more than 3% among the study population was identified between the cases and controls. Subjects with GD had a higher percentage of the genotype HLA-A*11:01-*11:01 (13.3% *vs.* 10.8%, *P* value = 3.15×10^-4^) and 02:07-11:01 (12.5% *vs.* 7.3%, *P* value = 1.83×10^-17^), HLA-B*40:01-*46:01 (12.5% *vs.* 8.3%, *P* value = 7.81×10^-10^) and *46:01-*46:01 (5.1% *vs.* 2.7%, *P* value = 4.73×10^-9^), and HLA-C*01:02-*01:02 (6.2% *vs.* 4.1%, *P* value = 8.67×10^-7^), *01:02-*03:04 (7.3% *vs.* 5.3%, *P* value = 2.80×10^-5^), and *01:02-*07:02 (12.6% *vs.* 8.8%, *P* value = 1.27×10^-9^) than the subjects without thyroid disorders (**Table 2**). All of the above associations passed the *P* value for multiple testing correction.

**Table 2:** HLA class I (HLA-A, -B, and -C) genotypes significantly associated with Graves’ disease in a Taiwanese population.

Locus	Genotype	Control	Hyperthyroid	*P*-value^a^	OR^b^	95%CI^b^
		N = 29,083	N = 2,047			
A	*11:01-*11:01	3131 (10.8%)	273 (13.3%)	3.15×10^-4^	1.28	1.12-1.46
	*11:01-*33:03	2644 (9.1%)	154 (7.5%)	0.017	0.81	0.69-0.96
	*02:07-*11:01	2132 (7.3%)	256 (12.5%)	1.83×10^-17^	1.81	1.57-2.08
	*24:02-*33:03	1539 (5.3%)	72 (3.5%)	4.60×10^-4^	0.65	0.51-0.83
	*24:02-*24:02	1290 (4.4%)	59 (2.9%)	0.001	0.64	0.49-0.83
B	*40:01-*46:01	2107 (8.3%)	225 (12.5%)	7.81×10^-10^	1.58	1.36-1.83
	*46:01-*46:01	681 (2.7%)	91 (5.1%)	4.73×10^-9^	1.93	1.54-2.42
	*40:01-*58:01	1927 (7.6%)	103 (5.7%)	0.004	0.74	0.62-0.91
C	*01:02-*01:02	1358 (4.1%)	142 (6.2%)	8.67×10^-7^	1.56	1.30-1.86
	*01:02-*03:04	1741 (5.3%)	166 (7.3%)	2.80×10^-5^	1.42	1.20-1.68
	*01:02-*07:02	2930 (8.8%)	287 (12.6%)	1.27×10^-9^	1.49	1.31-1.70
	*03:02-*03:04	1119 (3.4%)	59 (2.6%)	0.044	0.76	0.59-0.99

OR, odds ratio; CI, confidence interval; N, number.

a P value for chi square test. The P value threshold for multiple testing was set as 3.125×10^-3^ (=0.05/16), 1.515×10^-3^(=0.05/33), and 2.632×10^-3^ (=0.05/19) for HLA-A, -B, and -C, respectively.

b Results in the univariable logistic regression model. OR (95% CI) refer to the presence of the genotype.

With regard to the HLA class II subtypes (HLA-DP, -DQ, -DR), a total of 17 HLA genotypes were significantly associated with GD in the Taiwanese population. Subjects with GD showed a higher percentage of the genotype HLA-DPA1*02:02-*02:02 (45.8% *vs.* 39%, *P* value = 5.17×10^-13^), HLA-DPB1*02:01-*05:01 (19.8% *vs.* 15.9%, *P* value = 3.00×10^-6^), *02:02-*05:01 (4.4% *vs.* 7.8%, *P* value = 4.58×10^-13^), and *04:01-*05:01 (10.8% *vs.* 8.0%, *P* value = 9.00×10^-6^), HLA-DQA1*01:02-*03:02 (7.3% *vs* 5.5%, *P* value = 1.43×10^-4^) and *03:02-*05:05 (4.6% *vs* 3.5%, *P* value = 0.005), and HLA-DRB1*04:05-*09:01 (3.9% *vs* 2.9%, *P* value = 0.013), *09:01-*11:01 (4.2% *vs* 3.0%, *P* value = 0.006), *09:01-*15:01 (5.0% *vs.* 3.1%, *P* value = 9.00×10^-6^), and *09:01-*09:01 (6.0% *vs.* 3.5%, *P* value = 2.51×10^-8^) than the subjects without GD (**Table 3**). Among the above genotypes, HLA-DQA1*03:02-*05:05, and HLA-DRB1*04:05-*09:01 and *09:01-*11:01 did not pass the *P* value for multiple testing correction.

**Table 3:** HLA class II (HLA-DP, -DQ, and -DR) genotypes significantly associated with Graves’ disease in a Taiwanese population.

Locus	Genotype	Control	Hyperthyroid	*P*-value^a^	OR^b^	95%CI^b^
		N = 29,083	N = 2,047			
DPA1	*02:02-*02:02	16230 (39%)	1292 (45.8%)	5.17×10^-13^	1.33	1.23-1.43
	*01:03-*01:03	3293 (7.9%)	113 (4.0%)	5.04×10^-14^	0.49	0.40-0.59
	*02:01-*02:02	3697 (8.9%)	208 (7.4%)	0.007	0.82	0.71-0.95
	*01:03-*02:01	1627 (3.9%)	71 (2.5%)	1.99×10^-4^	0.64	0.50-0.81
DPB1	*02:01-*05:01	4914 (15.9%)	419 (19.8%)	3.00×10^-6^	1.30	1.17-1.46
	*02:02-*05:01	1354 (4.4%)	165 (7.8%)	4.58×10^-13^	1.84	1.56-2.18
	*04:01-*05:01	2479 (8.0%)	228 (10.8%)	9.00×10^-6^	1.38	1.20-1.60
DQA1	*03:02-*06:01	1163 (3.2%)	46 (1.9%)	2.47×10^-4^	0.58	0.43-0.78
	*01:02-*03:02	1974 (5.5%)	178 (7.3%)	1.43×10^-4^	1.36	1.16-1.60
	*03:02-*05:05	1264 (3.5%)	112 (4.6%)	0.005	1.33	1.09-1.62
	*01:02-*06:01	1201 (3.3%)	62 (2.5%)	0.035	0.76	0.59-0.98
DQB1	*03:01-*03:01	1991 (5.1%)	96 (3.6%)	0.001	0.69	0.56-0.86
DRB1	*04:05-*09:01	836 (2.9%)	74 (3.9%)	0.013	1.36	1.06-1.73
	*09:01-*12:02	1247 (4.3%)	53 (2.8%)	0.001	0.63	0.48-0.84
	*09:01-*11:01	872 (3.0%)	79 (4.2%)	0.006	1.39	1.10-1.76
	*09:01-*15:01	892 (3.1%)	94 (5.0%)	9.00×10^-6^	1.63	1.31-2.03
	*09:01-*09:01	1002 (3.5%)	113 (6.0%)	2.51×10^-8^	1.76	1.44-2.14

OR, odds ratio; CI, confidence interval; N, number.

a P value for chi square test. The P value threshold for multiple testing was set as 2.778×10^-3^, (=0.05/18), 1.667×10^-3^ (=0.05/30), and 2.5×10^-3^ (=0.05/20) for HLA-DP, -DQ, and -DR, respectively.

b Results in the univariable logistic regression model. OR (95% CI) refer to the presence of the genotype.

Furthermore, the association between the identified HLA genotypes and different comorbidities among subjects with GD was investigated. HLA-A*11:01-*33:03 (13.7% *vs.* 7.3%, *P* value = 0.042) and *02:07-*11:01 (2.7% *vs.* 12.9%, *P* value = 0.010), and HLA-B*40:01-*58:01 (12.5% *vs.* 5.5%, *P* value = 0.027) were significantly associated with heart disease. HLA-A*24:02-*24:02 (8.6% *vs.* 2.7%, *P* value = 0.014), and HLA-B*46:01-*46:01 (0% *vs.* 5.3%, *P* value = 0.046) were significantly associated with stroke. HLA-B*46:01-*46:01 (0% *vs.* 91%, *P* value = 0.013), DPA1*01:03-*01:03 (7.9% *vs.* 3.8%, *P* value = 0.012), and DPB1*02:01-*05:01 (12.0% *vs.* 20.3%, *P* value = 0.028) were significantly associated with hypertension. A higher percentage of HLA-DPA1*01:03-*01:03 (7.2% *vs.* 3.7%, *P* value = 0.006) and *01:03-*02:01 (4.9% *vs.* 2.3%, *P* value = 0.009) genotypes were identified among the subjects with GD that had diabetes than those with GD that did not have diabetes (**Table 4**). However, none of the above associations passed the *P* value for multiple testing correction.

**Table 4:** HLA class I and class II genotypes significantly associated with comorbidities among Taiwanese Graves’ disease patients.

Locus	Genotype	Heart Disease			Stroke			Hypertension			Diabetes		
		without	with	*P*^a^	without	with	*P*^a^	without	with	*P*^a^	without	with	*P*^a^
A	*11:01-*33:03	144 (7.3%)	10 (13.7%)	0.042									
	*02:07-*11:01	254 (12.9%)	2 (2.7%)	0.010									
	*24:02-*24:02				53 (2.7%)	6 (8.6%)	0.014						
B	*46:01-*46:01				91(5.3%)	0 (%)	0.046	91(5.3%)	0 (%)	0.013			
	*40:01-*58:01	95 (5.5%)	8 (12.5%)	0.027									
DPA1	*01:03-*01:03							101 (3.8%)	12 (7.9%)	0.012	94 (3.7%)	19 (7.2%)	0.006
	*01:03-*02:01										58 (2.3%)	13 (4.9%)	0.009
DPB1	*02:01-*05:01							405 (20.3%)	14 (12.0%)	0.028			

a P value for chi square test. The P value threshold for multiple testing was set as 1.43×10^-3^ (=0.05/35).

## Discussion

In the present study, we used a machine learning method, HIBAG, to predict the HLA subtypes and identified HLA class I (HLA-A*02:07, *11:01, *24:02 and *33:03, HLA-B*46:01, and HLA-C*01:02 and *12:02) and HLA class II (-DPA1*01:03, *02:01, and *02:02, -DPB1*05:01, -DQA1*03:02 and *06:01, -DQB1*03:01 and *03:03, and -DRB1*09:01 and *12:02) subtypes that were associated with GD in a Taiwanese population (**Tables S1, S2**). Moreover, the HLA subtypes HLA-A*11:01, HLA-B*46:01, HLA-DPA1*01:03, and HLA-DPB1*05:01 were associated with heart disease, stroke, diabetes, and hypertension among subjects with GD.

The HLA complex, which presents antigens for recognition by T cells, is known to be the most important region for humans with respect to infection and innate and adaptive immune systems. HLA genes are highly associated with drug reactions, organ transplantation, and human diseases, including autoimmune and infectious diseases. The HLA gene system is one of the most polymorphic regions of the human genome and has shown some ethnic differences in previous genetic association studies (12). In Caucasians, associations of HLA-DQA1*05:01 and -DQB1*03:01, -DRB1*03, and -DRB1*08 with GD and HLA-DRB1*03, -DRB1*04, -DRB1*08, -DQA1*03011/12, -DQA1*04:01, and -DQB1*0301/4 with Hashimoto Disease (HD) have been reported (13). In non-Caucasian populations, the HLA-B 46 allele was identified as a risk factor for GD in Asian populations, on the basis of the results of a meta-analysis (14). In the Japanese population, HLA-B*35:01, -B*46:01, -DRB1*14:03, -DQB1*06:04, and -DPB1*05:01 were found to be positively associated with GD (15), and HLA-A*02:07 and -DRB4 were found to be positively associated with HD (15). In the Chinese population, HLA-A*11:01/02, -B*46:01, -DRB1*15:01, -DRB1*16:02, -DQB1*03:02, and -DPB1*05:01 were found to be positively associated with GD (9). In the Korean population, HLA-DRB1*03:01:01, -DRB1*08:02:01, -DRB1*08:03, -DRB1*14:03:01, and -DRB1*16:02 were found to be positively associated with GD (16, 17). In the present study, we identified more HLA alleles related to GD compared with those identified in previous studies. In the haplotype analyses (**Table S3**), the haplotypes HLA-A*02:07-B*46:01-C*01:02, HLA-DPA1*02:02-DPB1*05:01, HLA-DRB1*09:01-DQA1*03:02-DQB1*03:03, and HLA-DRB1*12:02-DQA1*06:01-DQB1*03:01 showed a significant association with GD after considering multiple testing. The haplotype HLA-A*02:07-B*46:01-C*01:02 was also associated with GD in Korean children (18).

Moreover, it is well known that thyroid hormone dysfunction is devastating to the heart and cardiovascular system due to the alterations in cardiac contractility, myocardial oxygen consumption, cardiac output, blood pressure, and systemic vascular resistance (19). Cardiovascular mortality is increased in hyperthyroidism (19–22) and the severity of hyperthyroidism-associated cardiomyopathy is second only to that of hyperthyroidism crisis, which is an important cause of death in hyperthyroidism patients (23, 24). However, only a few studies have investigated the effects of HLA variants on vascular diseases or metabolic diseases among patients with hyperthyroidism. Several studies have demonstrated the association between Moyamoya disease (MMD), a progressive cerebrovascular disease with unknown etiology and allelic and haplotypic differences of HLA, including differences in both HLA class I and class II alleles (e.g., HLA-A*24, HLA-B*54, HLA-DRB1*04:05, *04:10, and HLA-DQB1*04:01) in multiple cohorts of East Asians (25–27), as well as in Europeans (28). In a meta-analysis that included three cohorts, the absence of HLA-DRB5 was associated with an increased risk of type 2 diabetes (P = 0.001). In contrast, HLA-DQB*06:02 and HLA-DQA*01:02 were shown to have protective effects against type 2 diabetes (P = 0.005 and 0.003, respectively) (29).

Our study had several strengths. We used the HIBAG algorithm, an ensemble haplotype-based classifier, to impute HLA types using attribute BAGging, which makes predictions by averaging HLA-type posterior probabilities over an ensemble of classifiers that are built on the basis of different samples (30, 31). This HIBAG method has been successfully used to investigate the association between HLA alleles and rheumatoid arthritis in a Taiwanese population (31). In the present study, by using these imputed HLA alleles, we identified several HLA alleles that were notably associated with GD in a population for which genome-wide SNP array data were available. Another strength of our study was that we extracted data from the EMR, which provides comprehensive information regarding the diagnosis of diseases, medicine use, and laboratory data for each individual and clearly defines the cases and controls. Moreover, the sample size in the present study was large, which increased its statistical power. However, our study also has a few limitations: firstly, this study was performed based on the EMR from a single medical center, which may limit the generalizability of our findings; secondly, no information regarding lifestyle-associated factors, such as physical activity, alcohol use, and tobacco smoking on the EMR, were analyzed, due to which we could not exclude the potential residual confounding effects of these factors.

In conclusion, we used a powerful imputation method, HIBAG, to predict the HLA subtypes among populations with available genome-wide SNP array data and linked EMR data to identify several HLA alleles that were markedly associated with GD and its comorbidities, including heart disease, hypertension, and diabetes; to the best of our knowledge, our study is the first to report the influence of different HLA subtypes on GD-associated comorbidities. The functional considerations for the relevant HLA alleles and the implications of our study’s findings could be study in future research on this field.

## Data Availability Statement

The original contributions presented in the study are included in the article/**Supplementary Material**, further inquiries can be directed to the corresponding authors.

## Ethics Statement

The studies involving human participants were reviewed and approved by China Medical University Hospital. The patients/participants provided their written informed consent to participate in this study.

## Author Contributions

TYW and F-JT conceived the project and designed the experiments. C-FC and Y-PC performed HLA imputation. W-LL, Y-WC, and T-YL performed the data analysis. W-LL wrote the original manuscript. W-LL, T-YL, and S-YC revised the manuscript with input from all authors. All authors contributed to the article and approved the submitted version.

## Funding

This study was supported by China Medical University and China Medical University Hospital (CMUH110-MF-71 and DMR-111-137), and the Ministry of Science and Technology (MOST 109-2314-B-039-033-MY2).

## Conflict of Interest

The authors declare that the research was conducted in the absence of any commercial or financial relationships that could be construed as a potential conflict of interest.

## Publisher’s Note

All claims expressed in this article are solely those of the authors and do not necessarily represent those of their affiliated organizations, or those of the publisher, the editors and the reviewers. Any product that may be evaluated in this article, or claim that may be made by its manufacturer, is not guaranteed or endorsed by the publisher.
