# Supplementary material for: Analysis of HLA Variants and Graves’ Disease and Its Comorbidities Using a High Resolution Imputation System to Examine Electronic Medical Health Records
Source: Front Endocrinol (Lausanne). 2022 Mar 7;13:842673. doi: 10.3389/fendo.2022.842673 (PMC8936090; doi:10.3389/fendo.2022.842673)
Supplement: Supplementary file 1 [file DataSheet_1.pdf]

Table S1 HLA class I (HLA-A,-B, and -C) allele significantly associated with Graves' disease in a Taiwan Han population.

Locus	Allele	Allele frequency [e]						
		Taiwan Han population [a] N = 710	Taiwan Han population [b] N = 504	Control N = 58,166	Case N = 4,094	*P* value [c]	OR [d]	95% CI [d]
A	*01:01	0.009	0.0040	349 (0.6%)	7 (0.2%)	$4.33\times10^{-4*}$	0.28	0.13-0.60
	*02:01	0.1040	0.0920	3060 (5.3%)	181 (4.4%)	0.019	0.83	0.72-0.97
	*02:07	0.0990	0.1120	6585 (11.3%)	700 (17.1%)	$1.05\times10^{-28*}$	1.62	1.48-1.76
	*11:01	0.2750	0.3260	19111 (32.9%)	1492 (36.4%)	$2.00\times10^{-6*}$	1.17	1.10-1.25
	*24:02	0.1700	0.1580	11444(19.7%)	655 (16.0%)	$9.19\times10^{-9*}$	0.78	0.71-0.85
	*26:01	0.0250	0.0240	1670 (2.9%)	93 (2.3%)	0.025	0.79	0.64-0.97
	*30:01	0.0130	0.0120	947 (1.6%)	41 (1.0%)	0.002	0.61	0.45-0.84
	*32:01	0.0040	0.0050	230 (0.4%)	8 (0.2%)	0.045	0.49	0.24-1.00
	*33:03	0.1040	0.1180	8053 (13.8%)	471 (11.5%)	$2.60\times10^{-5*}$	0.81	0.73-0.89
B	*13:01	0.0530	0.0560	3753 (7.4%)	314 (8.7%)	0.003	1.20	1.06-1.35
	*13:02	0.0150	0.0170	924 (1.8%)	40 (1.1%)	0.002	0.61	0.44-0.83
	*15:25		0.0040	447 (0.9%)	17 (0.5%)	0.010	0.53	0.33-0.87
	*27:04	0.0350	0.0260	1821 (3.6%)	83 (2.3%)	$5.30\times10^{-5*}$	0.63	0.51-0.79
	*37:01	0.0080	0.0030	219 (0.4%)	6 (0.2%)	0.017	0.39	0.17-0.87
	*46:01	0.1260	0.1360	8201 (16.2%)	840 (23.4%)	$4.97\times10^{-29*}$	1.58	1.46-1.71
	*52:01	0.0110	0.0100	586 (1.2%)	21 (0.6%)	0.002	0.50	0.33-0.78

	*58:01	0.0980	0.1060	7139 (14.1%)	428 (11.9%)	$2.72\times10^{-4*}$	0.83	0.74-0.92
C	*01:02	-	0.2120	13617 (20.5%)	1240 (27.3%)	$4.42\times10^{-27*}$	1.45	1.36-1.55
	*03:02	-	0.1050	8252 (12.5%)	485 (10.7%)	$4.03\times10^{-4*}$	0.84	0.76-0.93
	*04:03	-	0.0190	1184 (1.8%)	56 (1.2%)	0.006	0.69	0.52-0.90
	*06:02	-	0.0200	1518 (2.3%)	64 (1.4%)	$9.8\times10^{-5*}$	0.61	0.47-0.78
	*08:01	-	0.0810	4316 (6.5%)	257 (5.7%)	0.023	0.86	0.76-0.98
	*12:02	-	0.0400	2993 (4.5%)	135 (3.0%)	$9.08\times10^{-7*}$	0.65	0.54-0.77
	*15:02	-	0.0410	2625 (4.0%)	135 (3.0%)	0.001^{*}	0.74	0.62-0.88
	*16:02	-	0	17 (0.025%)	5 (0.1%)	0.002	4.29	1.58-11.64

[a] From S-H Wen et al. (2008) *Human Immunology* 69:430

[b] From P-L Chen et al. (2011) *PLoS ONE* 6(1):e16635

[c] *P* value for chi square test. *The *P* value is smaller than the threshold for multiple testing which was set at 3.125×10^{-3} (=0.05/16), 1.515×10^{-3}(=0.05/33), and 2.632×10^{-3} (=0.05/19) for HLA-A, -B, and -C, respectively.

[d] Results in the univariable logistic regression model. OR (95% CI) refer to the presence of the allele.

[e] Total number of copies of the allele in the population sample (Alleles / 2n) in decimal format.

Table S2 HLA class II (HLA-DP,-DQ, and -DR) allele significantly associated with Graves' disease in a Taiwan Han population.

Locus	Allele	Allele frequency [e]						
		Taiwan Han population [a]	Taiwan Han population [b]	Control	Case	*P* value [c]	OR [d]	95% CI [d]
		N = 710	N = 504	N = 58166	N = 4094			
DPA1	*01:03	-	-	23245 (27.9%)	1326 (23.5%)	$1.03\times10^{-12*}$	0.80	0.75-0.85
	*02:01	-	-	5848 (7.0%)	295 (5.2%)	$3.00\times10^{-7*}$	0.73	0.65-0.83
	*02:02	-	-	51977 (62.4%)	3896 (69.1%)	$6.18\times10^{-24*}$	1.35	1.27-1.43
	*04:01	-	-	2204 (2.6%)	119 (2.1%)	0.015	0.79	0.66-0.96
DPB1	*02:02	-	0.0850	3747 (6.1%)	289 (6.8%)	0.047	1.13	1.00-1.28
	*03:01	-	0.0540	2781 (4.5%)	152 (3.6%)	0.005	0.79	0.67-0.93
	*04:02	-	0.0160	927 (1.5%)	37 (0.9%)	0.001^{*}	0.58	0.42-0.80
	*05:01	-	0.4370	31417 (50.9%)	2357 (55.7%)	$1.70\times10^{-9*}$	1.21	1.14-1.29
	*09:01	-	0.0160	856 (1.4%)	26 (0.6%)	$2.30\times10^{-5*}$	0.44	0.30-0.65
	*13:01	-	0.0520	4047 (6.6%)	235 (5.6%)	0.010	0.84	0.73-0.96
	*17:01	-	0.0120	688 (1.1%)	24 (0.6%)	0.001^{*}	0.51	0.34-0.76
	*19:01	-	0.0130	951 (1.5%)	42 (1.0%)	0.005	0.64	0.67-0.87
	*21:01	-	0.0190	991 (1.6%)	35 (0.8%)	$7.50\times10^{-5*}$	0.51	0.36-0.72
	*104:01	-		75 (0.1%)	0 (0%)	0.015	-	
DQA1	*01:02	-	-	12745 (17.6%)	936 (19.1%)	0.007	1.11	1.03-1.19

	*01:04	-	-	5396 (7.5%)	319 (6.5%)	0.016	0.87	0.77-0.97
	*01:05	-	-	753 (1.0%)	20 (0.4%)	$1.7\times10^{-5*}$	0.39	0.25-0.61
	*02:01	-	-	1480 (2.0%)	61 (1.2%)	$1.12\times10^{-4*}$	0.61	0.47-0.78
	*03:02	-	-	11731 (16.2%)	950 (19.4%)	$4.44\times10^{-9*}$	1.25	1.16-1.34
	*03:03	-	-	4331 (6.0%)	330 (6.7%)	0.030	1.14	1.01-1.28
	*06:01	-	-	7183 (9.9%)	262 (5.4%)	$1.04\times10^{-25*}$	0.51	0.45-0.58
DQB1	*02:02	-	0.0240	1522 (1.9%)	59 (1.1%)	$1.20\times10^{-5*}$	0.56	0.43-0.73
	*03:01	-	0.2120	17692 (22.6%)	1067 (20.0%)	$6.00\times10^{-6*}$	0.85	0.80-0.91
	*03:03	-	0.1710	12465 (15.9%)	993 (18.6%)	$4.10\times10^{-7*}$	1.20	1.12-1.29
	*04:01	-	0.0690	5282 (6.8%)	405 (7.6%)	0.021	1.13	1.02-1.26
	*05:01	-	0.0250	1788 (2.3%)	82 (1.5%)	$3.18\times10^{-4*}$	0.67	0.53-0.83
	*05:02	-	0.0930	7950 (10.2%)	616 (11.5%)	0.002	1.15	1.06-1.26
	*05:03	-	0.0490	3318 (4.2%)	184 (3.4%)	0.005	0.80	0.69-0.94
	*06:09	-	0.0220	1701 (2.2%)	50 (0.9%)	$9.11\times10^{-10*}$	0.43	0.32-0.56
DRB1	*01:01	0.0060	0.0050	306 (0.5%)	9 (0.2%)	0.014	0.45	0.23-0.86
	*07:01	0.0230	0.0280	1422 (2.5%)	61 (1.6%)	0.001^{*}	0.65	0.50-0.84
	*09:01	0.1490	0.1660	10553 (18.3%)	859 (22.6%)	$4.56\times10^{-11*}$	1.30	1.20-1.41
	*10:01	0.0170	0.0070	717 (1.2%)	16 (0.4%)	$6.00\times10^{-6*}$	0.34	0.20-0.55
	*12:01	0.0410	0.0330	2098 (3.6%)	162 (4.3%)	0.049	1.18	1.00-1.39
	*12:02	0.1010	0.0860	6827 (11.9%)	249 (6.6%)	$3.92\times10^{-23*}$	0.52	0.46-0.60
	*13:02	-	0.0220	1602 (2.8%)	49 (1.3%)	$3.71\times10^{-8*}$	0.46	0.34-0.61

	*14:05	0.0210	0.0220	1181 (2.1%)	60 (1.6%)	0.046	0.77	0.59-1.00
	*15:01	0.0890	0.0680	5060 (8.8%)	387 (10.2%)	0.003	1.18	1.06-1.31

[a] From S-H Wen et al. (2008) *Human Immunology* 69:430

[b] From P-L Chen et al. (2011) *PLoS ONE* 6(1):e16635

[c] *P* value for chi square test. * The *P* value is smaller than the threshold for multiple testing which was set at 2.778×10^{-3}, (=0.05/18), 1.667×10^{-3} (=0.05/30), and 2.5×10^{-3} (=0.05/20) for HLA-DP, -DQ, and -DR, respectively.

[d] Results in the univariable logistic regression model. OR (95% CI) refer to the presence of the allele.

[e] Total number of copies of the allele in the population sample (Alleles / 2n) in decimal format.

Table S3 HLA class I and class II haplotypes significantly associated with Graves' disease in a Taiwan Han population.

	Control	Case	*P* value [a]	OR [b]	95% CI [b]
A*11:01-B*46:01-C*01:02	356 (1.1%)	44 (1.8%)	0.001	1.70	1.24-2.34
A*24:02-B*46:01-C*01:02	177 (0.5%)	5 (0.2%)	0.029	0.39	0.16-0.94
A*02:07-B*46:01-C*01:02	1730 (5.1%)	190 (7.7%)	$3.72\times10^{-8*}$	1.54	1.32-1.80
DPA1*02:02-DPB1*05:01	27912 (45.9%)	2122 (51.1%)	$5.05\times10^{-11*}$	1.23	1.16-1.32
DRB1*09:01-DQA1*03:02-DQB1*03:03	2162 (4.2%)	235 (6.8%)	$1.03\times10^{-13*}$	1.69	1.47-1.94
DRB1*12:02-DQA1*06:01-DQB1*03:01	2118 (4.1%)	62 (1.8%)	$2.85\times10^{-11*}$	0.43	0.33-0.56
DRB1*12:02-DQA1*06:01-DQB1*03:03	1148 (2.2%)	46 (1.3%)	0.001	0.60	0.45-0.81

[a] *P* value for chi square test. *The *P* value is smaller than the threshold for multiple testing which was set at 9.8×10^{-4} (=0.05/51).

[b] Results in the univariable logistic regression model. OR (95% CI) refer to the presence of the haplotype.
